# Functional Effect of Polymorphisms in the Promoter of *TNFAIP3* (A20) in Acute Pancreatitis in the Han Chinese Population

**DOI:** 10.1371/journal.pone.0103104

**Published:** 2014-07-22

**Authors:** Yugang Liu, Gang Dan, Lijuan Wu, Guangyu Chen, Ailin Wu, Ping Zeng, Wanqing Xu

**Affiliations:** 1 Center of Laboratory Medicine, Chengdu Military General Hospital, Chengdu, P.R. China; 2 Department of General Surgery, Chengdu Military General Hospital, Chengdu, P.R. China; University of Chicago, United States of America

## Abstract

**Background:**

The zinc finger protein A20 is an important negative regulator of inflammation; polymorphisms in the corresponding gene, *TNFAIP3*, have been reported to be associated with several inflammation diseases. However, only a few studies have focused on the relationship between *TNFAIP3* polymorphisms and acute pancreatitis (AP).

**Methods:**

We enrolled 201 healthy controls and 190 acute pancreatitis patients (including 47 systemic inflammatory response syndrome patients) for this study and used DNA sequencing to investigate polymorphisms in the *TNFAIP3* promoter. The functional effects of these variants on transcriptional activity, A20 expression, NF-κB activity, and TNF-α and IL-1β levels, after in vitro lipopolysaccharide stimulation, were assessed.

**Results:**

Two SNPs (rs59693083 and rs5029924) in the *TNFAIP3* promoter were selected based on bioinformatic analysis. Neither of these SNPs was associated with susceptibility to AP; however, acute pancreatitis patients who possessed the T allele of rs5029924 were more likely to experience systemic inflammatory response syndrome. Moreover, rs5029924 was found to affect *TNFAIP3* promoter activity. After lipopolysaccharide stimulation, the expression of A20 protein significantly decreased, while the activity of NF-κB and the production of TNF-α and IL-1β significantly increased in whole blood leukocytes from subjects with the T allele.

**Conclusion:**

The rs5029924 polymorphism in the *TNFAIP3* promoter may alter the risk of systemic inflammatory response syndrome in acute pancreatitis patients by influencing the expression of A20 protein.

## Introduction

Acute pancreatitis (AP) is a syndrome of sudden-onset pancreatic inflammation characterized by upper abdominal pain, nausea, emesis, elevation of serum amylase and lipase levels, and changes noted upon abdominal imaging [1]. Following global lifestyle and behavior changes, the incidence of AP has increased markedly in recent years [2]. The inflammatory process in AP is usually limited to the pancreas or spreads only to regional tissues, so that the majority of patients experience mild disease. However, in about 20% of patients, the inflammatory process involves more than the pancreas, leading to a severe course; almost one-third of these patients progress to systemic inflammatory response syndrome (SIRS), and, subsequently, develop multi-organ dysfunction syndrome (MODS) [3], [4], which is the main cause of death in critically ill patients. Therefore, preventing SIRS/MODS is crucial in the treatment of patients with AP. Nevertheless, the specific mechanisms underlying the transition from local pancreatic inflammation to SIRS have not yet been elucidated clearly.

Individual differences in the occurrence, transition, and development of AP indicates that genetic background may play an important role in AP. The pancreatic protease/anti-protease system and inflammatory cytokines play pivotal roles in the pathogenesis of AP [5]; hence, most previous studies have mainly focused on polymorphisms occurring in genes related to these systems [6]–[9]. It has been reported that the anti-inflammatory response also plays an important role in AP, but limited studies have investigated the relationship between polymorphisms in genes encoding anti-inflammatory molecules and AP.

A20, also known as the TNF-α-induced protein 3 (TNFAIP3), is a zinc finger protein that was first identified from a gene, *TNFAIP3*, that was induced by TNF-α in human umbilical vein endothelial cells [10]; it is now known that A20 is widely expressed in a variety of human cells. As a dual ubiquitin-editing enzyme, A20 plays a central and ubiquitous role in negatively regulating the activity of the transcription factor NF-κB and the proinflammatory gene expression that is triggered by signaling from Toll-like receptors (TLRs) and NOD2 [11], [12]. The inhibitory effect is mediated by inactivation of several proteins that are critical to NF-κB signaling, such as RIP1/2, TRAF2/6, NEMO, and TAK1, through ubiquitination or de-ubiquitination [13]–[16].

A20 plays a critical role in the pathogenesis of inflammatory diseases [17].

Promoters are gene regions that are important for determining disease susceptibility and gene regulation [18], but it has not yet been clarified whether polymorphisms in the *TNFAIP3* promoter are associated with AP susceptibility, or progression to SIRS. Therefore, we sought to determine the association of potential polymorphisms with susceptibility to AP and subsequent SIRS in a case-control study involving the Chinese Han population. Additionally, we investigated the functional effect of these polymorphisms on the promoter activity and expression of *TNFAIP3*.

## Materials and Methods

### 2.1. Subjects

The protocols were approved by the Ethics Committee of Chengdu Military General Hospital, and informed written consent was obtained from all patients and controls before their enrollment in the study.

We enrolled 190 patients who had AP and had been admitted to the Chengdu Military General Hospital, Chengdu, China, between January 2012 and October 2013. All patients were followed up until discharge. Among them, 61 patients had severe AP (SAP), and 47 patients developed persistent SIRS (>48 h). The Acute Physiology and Chronic Health Evaluation (APACHE II) score [19] was calculated for all patients. Controls included 201 healthy individuals who were recruited in the medical examination center of Chengdu Military General Hospital. The exclusion criteria were as follows: genetic relationships with other subjects, any chronic underlying disease, or other acute diseases within the previous month, age >70 y or <18 y, and pregnancy.

AP and SAP were diagnosed according to the criteria defined by the summary of the Atlanta International Symposium on AP in 1992 [20]. The diagnosis of AP was made when two or more of the following three criteria were met: abdominal pain, amylase and/or lipase levels elevated to at least three times greater than the upper limit of normal, and abdominal imaging indicative of AP. SIRS was defined according to the SCCM/ESICM/ACCP/ATS/SIS criteria [21].

### 2.2. Bioinformatics analysis

The full sequence of human *TNFAIP3* (Genbank accession no.: NC_000006.11) is located on chromosome 6, position 138188325–138204451, and its 5′-flanking region includes 2 kb upstream of the transcription start site (www.ncbi.nlm.nih.gov/genbank/). To determine the possible functionality of SNPs selected from the 5′-flanking region of *TNFAIP3*, TFSEARCH (www.cbrc.jp/research/db/TFSEARCH.html and genome.ad.jp) was used to analyze the effect of these SNPs on potential transcription factor binding sites. Genetic variation data for the promoter of the gene were obtained from the 1000 genomes project (www.1000genomes.org) for Han Chinese in Beijing (CHB).

### 2.3. DNA extracting and genotyping

Genomic DNA was extracted from the whole blood of participants with a DNA Blood Extraction kit (Sangon, Shanghai, China) in accordance with the manufacturer's protocol. The DNA extracts were stored at −20°C until required for analysis. Two sets of PCR were used to analyze the SNPs. Each PCR was performed in a total reaction volume of 50 µL, containing 50 ng of genomic DNA, 0.5 µL PrimeSTAR HS DNA Polymerase (Takara, Dalian, China), 4 µL of 2.5 mM dNTP, 1 µL of each primer (10 µM), and 25 µL 2× PrimeSTAR Buffer (Takara). For rs59693083, we used primers 5′-GGCAAGATGACAAAGCAGTT-3′ and 5′-AATGCGGCTTCAGAGGTAG-3′, along with the following PCR conditions: 3 min at 95°C, followed by 35 cycles, each consisting of 30 s at 94°C (denaturation), 15 s at an annealing temperature of 53°C, and 30 s at 72°C (extension). For rs5029924, primers 5′-GTAAAAACGCCCTAAGACTCC-3′ and 5′-ATTCAAAGCAGCATTTCAGC-3′ were used with the following cycling conditions: 3 min at 95°C, followed by 35 cycles, each consisting of 30 s at 94°C, 15 s at 53°C, and 30 s at 72°C. Both PCRs concluded with a 5-min extension at 72°C.

PCR products were routinely purified and directly sequenced with the Applied Biosystems BigDye terminator chemistry 3.1 system (Applied Biosystems, Foster City, CA, USA), and the sequences were resolved with an ABI 3730 Genetic Analyzer. Sequences were aligned using DNAStar (DNAStar, Madison, WI, USA), and two researchers interpreted the results.

### 2.4. *TNFAIP3* promoter activity assay

The possible effect of rs5029924 on *TNFAIP3* promoter activity was investigated using a reporter gene assay system. Two plasmid constructs were prepared by inserting a 1015-bp sequence (−924 to +91 of *TNFAIP3*), encompassing the investigated single nucleotide polymorphisms (SNPs), into a promoterless pGL3-Basic vector (Promega, Madison, WI, USA), which contained either the −827C allele or the −827T allele. The constructed vectors (2 µg DNA) were individually and transiently transfected into human U937 cells, using FuGENE6 reagent (Roche Molecular Biochemicals, Indianapolis, IN, USA). Cells were harvested 20 h later; firefly and *Renilla* luciferase assays were performed using the dual luciferase assay kit (Promega, Madison, WI, USA) as per the manufacturers' instructions. Experiments were performed in triplicate on at least three separate occasions. Results are expressed as fold-increase in luciferase/*Renilla* activity of the *TNFAIP3* promoter construct vectors compared with that of pGL3-Basic.

### 2.5. In vitro lipopolysaccharide (LPS) stimulation of whole blood

We used peripheral whole blood from healthy subjects and stimulated these samples with LPS to simulate SIRS. Whole blood was collected from 60 healthy subjects, with different genotypes at the *TNFAIP3* promoter, and immediately mixed the blood 1∶1 (vol/vol) with RPMI-1640 culture medium. These samples were then incubated with 100 ng/mL *Escherichia coli* 055:B5 LPS (Sigma–Aldrich, St Louis, MO, USA) at 37°C for 4 h [22]. After incubation, supernatants and cell pellets were harvested by centrifugation and stored at −80°C until required for use, within 48 h.

### 2.6. RNA purification and mRNA expression analysis

Total RNA was extracted from peripheral blood samples using a QIAamp RNA Blood Mini kit (Qiagen, Valencia, CA, USA). Aliquots of RNA (100 ng) were used for cDNA synthesis with a PrimeScript RT Reagent kit (Takara, Dalian, China), according to the manufacturer's protocol. The synthesized cDNA was used for real-time PCR, which was performed using a SYBR Premix ExTaq kit (Takara, Dalian, China), on an ABI 7900HT system (Applied Biosystems, Foster City, CA, USA). The primers for the A20 gene were as follows: forward 5′-AAGCCGGCTGCGTGTATTTTG-3′ and reverse 5′-GTCTTCGGGGGCAGGCTCACC-3′. The primers for the endogenous control gene (*ACTB*, encoding β-actin) were as follows: forward 5′-CTACAATGAGCTGCGTGTGG-3′, and reverse 5′-AAGGAAGGCTGGAAGAGTGC-3′. We carried out an initial denaturation step at 95°C for 30 s, followed by 40 cycles of PCR (95°C for 10 s, 60°C for 40 s). *TNFAIP3* mRNA expression levels were normalized to those of *ACTB*.

### 2.7. Intracytoplasmic A20 protein level analysis

Intracytoplasmic levels of A20 protein in peripheral blood leucocytes were determined by flow cytometry. Cell pellets (50 µL) were incubated with 10 µL CD45-PC5 (Immunotech, Marseille, France) antibody in the dark for 60 min. Then, Intraprep Permeabilization Reagents 1 and 2 were added (Beckman Coulter, Inc., Brea, CA, USA), after which samples were incubated first with 20 µL of A20 monoclonal antibody, and then with 20 µL goat-anti-mouse IgG-FITC (Active Motif, Carlsbad, CA, USA), in the dark, for 30 min each time. Samples were then mixed with 500 µL phosphate-buffered saline (PBS) prior to flow cytometric analysis.

A Coulter Epics XL3 flow cytometer (Beckman Coulter, Inc., Brea, CA, USA) was used to measure the mean fluorescent intensity (MFI) of FITC by gating on CD45^+^; the negative control was detected simultaneously. Intracytoplasmic A20 protein levels were represented by the MFI. Coulter System IITM software (Beckman Coulter, Inc., Brea, CA, USA) was used for data analysis. Instrument quality control was performed daily using homeotype controls and Immuno-Trol (Beckman Coulter, Inc., Brea, CA, USA) to allow consistent determinations over the course of the study.

### 2.8. NF-κB activity assay

We evaluated the ability of NF-κB to bind to the relevant DNA sequence in vitro using an electrophoretic mobility shift assay (EMSA). Peripheral blood mononuclear cells (PBMCs) were derived from three participant samples in the same pool of above 60 healthy subjects by means of the Ficoll gradient density centrifugation method. Nuclear proteins from PMBCs were extracted using a Nuclear–Cytosol Extraction kit (Applygen Technologies, Inc., Beijing, China). EMSA was performed using biotin-labeled double-stranded DNA oligonucleotides binding to the NF-κB in triplicate. The oligonucleotides for EMSA were 5′-biotin-AGTTGAGGGGACTTTCCCAGGC-3′ (forward), and 5′-biotin-GCCTGGGAAAGTCCCCTCAACT-3′ (reverse). Reactions contained 1.5 µL 10× buffer (10 mM Tris [pH 7.8], 50 mM NaCl, 1 mM DTT, 1 mM EDTA, 5% glycerol), 1.0 µL Poly dI∶dC, 0.5 µL biotin-labeled probe, and 3 µg nuclear extract. Reaction mixtures were incubated at room temperature for 30 min, loaded onto 5% 0.25× TBE gels, and electrophoresed at 120 V for 1 h. Gels were then electrotransferred and chemiluminescence was detected by streptavidin conjugated to horseradish peroxidase, and integral optical density (IOD) was recorded.

Competition experiments were performed by adding 100× unlabeled oligonucleotides to the reaction mixtures prior to addition of the radiolabelled probe as indicated.

### 2.9. TNF-α and IL-1β levels

The concentrations of TNF-α and IL-1β in supernatant plasm from above 60 healthy subjects were detected, in duplicate, using the appropriate enzyme-linked immunosorbent assay (ELISA) kits (R&D Inc., Minneapolis, MN, USA), according to the manufacturer's protocol. A MK3 enzyme-labeled instrument (Thermo Fisher Scientific, Waltham, MA, USA) was used for detecting optical density.

### 2.10. Statistical analysis

The demographic variables between different groups were compared by chi-square test for categorical data and by *t*-test for quantitative data. The genotype data were analyzed for deviations from Hardy-Weinberg equilibrium. The differences in allele and genotype distributions were compared by the chi-square test or Fisher's exact test. The odds ratio (OR) and 95% confidence interval (95% CI) were calculated. Linkage disequilibrium (LD) analysis between the pair of SNPs was estimated with the D′ and r^2^ scores. Relative *TNFAIP3* mRNA expression data was calculated using the 2^−ΔΔCT^ method [23]. Differences in the IOD of NF-κB complex, the relative mRNA expression and in the MFI, TNF-α, and IL-1β levels between individuals harboring the different genotypes were evaluated by independent sample *t*-test. SPSS version 16 (SPSS, Inc., Chicago, IL, USA) was used for all statistical analysis. Two-sided *p*-values <0.05 were considered to be statistically significant.

## Results

### 3.1. Characteristics of the study subjects

The relevant characteristics of the subjects are shown in Table 1. The etiologies of AP were ascertained in 135 patients, of whom 47 (24.7%) developed SIRS. The most common cause of AP was biliary disease (83/135, 43.7%), followed by idiopathic origin (45/135, 23.7%), alcoholic consumption (33/135, 17.4%), and post-endoscopic retrograde cholangiopancreatography procedure (ERCP) (16/135, 8.4%); in a few, the basis was hypertriglyceridemia (11/135, 5.8%) and other causes (2, 1.1%). Patients with SIRS had higher APACHE II scores than patients without SIRS (*p*<0.001). There were no other significant differences in the demographics (including mean age and gender distribution) between AP patients and healthy controls, or SIRS and non-SIRS AP.

**Table 1:** Demographics and clinical characteristics of all subjects.

	Healthy controls	Non-SIRS AP	SIRS	*p*-value
Number	201	143	47	
Age (years)	50.55±11.91	51.87±12.63	53.68±10.17	0.057
Gender (males/females)	135/66	85/58	33/14	0.236
APACHE II	—	4.31±2.04	16.26±6.94	<0.001
Etiology				
Biliary	—	62	21	
Alcohol	—	34	11	
Idiopathic	—	24	9	
Post-ERCP	—	14	2	
Hypertriglyceridemia	—	7	4	
Drugs	—	1	0	
Tumors	—	1	0	

AP indicates acute pancreatitis; SIRS indicates systemic inflammatory response syndrome.

### 3.2. SNP selection

SNP selection was based on the published data [24], [25], minor allele frequency (MAF) in CHB and location within the possible functional sequence. Data from the 1000 genomes project indicated that there were 22 SNPs within the 5′-flanking region of *TNFAIP3*, two of which, −1793A/G (rs59693083) and −827C/T (rs5029924), had a MAF of more than 5% in the CHB. Bioinformatics-based transcription factor analysis revealed that rs5029924 was within a sequence similar to GATA-1 and GATA-2 binding sites, suggesting that this genetic variation might affect the transcription of *TNFAIP3*. Accordingly, rs5029924 was selected as the maximally informative SNP, with rs59693083 as a possible influential polymorphism.

### 3.3. Analysis of association of *TNFAIP3* promoter polymorphisms with susceptibility to AP and risk of SIRS

Table 2 shows the allele and genotype frequencies of AP patients and control individuals. The genotype distributions in the control and case groups were in Hardy–Weinberg equilibrium (*p*>0.05). Genotype frequencies in the controls were in accordance with those derived from samples of the CHB from the 1000 genomes project. There were no differences in the allele or genotype distribution among SAP patients, AP patients and controls for either rs59693083 or rs5029924 (all *p*>0.05). As shown in Table 3, when patients were stratified according to etiology (biliary, alcoholic, and other etiology) AP, there were also no difference in genotype or allele frequencies when compared to the controls (all *p*>0.05).

**Table 2:** Genotype and allele frequencies of variants in the *TNFAIP3* promoter in healthy controls and AP patients.

	Healthy controls	AP patients	SAP patients	*p*-value	OR	95% CI
rs5029924						
Genotypes						
CC	155 (77.1%)	135 (71.1%)	41(67.2%)			
CT+TT	46 (22.9%)	55 (28.9%)	20(32.8%)	0.171^a^	1.373^a^	0.871–2.163^a^
				0.119^b^	1.644^b^	0.877–3.079^b^
CT	44 (21.9%)	52 (27.3%)	19(31.1%)			
TT	2 (1.0%)	3 (1.6%)	1(1.7%)			
Alleles						
C	354 (88.1%)	322 (84.7%)	101(82.8%)			
T	48 (11.9%)	58 (15.3%)	21(17.2%)	0.175^a^	1.328^a^	0.881–2.004^a^
				0.131^b^	1.533^b^	0.877–2.680^b^
rs59693083						
Genotypes						
AA	139 (69.2%)	129 (67.9%)	42(68.9%)			
AG+GG	62 (30.8%)	61 (32.1%)	19(31.1%)	0.789^a^	1.060^a^	0.692–1.625^a^
				0.495^b^	1.228^b^	0.681–2.215^b^
AG	56 (27.9%)	56 (29.5%)	15(24.6%)			
GG	6 (2.9%)	5 (2.6%)	4(6.5%)			
Alleles						
A	334 (83.1%)	314 (82.6%)	99(81.1%)			
G	68 (16.9%)	66 (17.4%)	23(18.9%)	0.867^a^	1.032^a^	0.712–1.498^a^
				0.835^b^	0.943^b^	0.541–1.643^b^

AP indicates acute pancreatitis; SAP indicates severe AP; CI indicates confidence interval; OR indicates odds ratio;

a indicates AP patients compare with healthy controls;

b indicates SAP patients compare with healthy controls.

**Table 3:** Genotype and allele frequencies of *TNFAIP3* promoter variants in different individuals with different AP etiologies.

	Healthy controls	Biliary	Alcohol	Others	*p*-value
rs5029924					
Genotypes					
CC	155 (77.1%)	59 (71.1%)	33 (73.3%)	43 (69.4%)	
CT+TT	46 (22.9%)	24 (28.9%)	12 (26.7%)	19 (30.6%)	0.554
CT	44 (21.9%)	23 (27.7%)	11 (24.5%)	18 (29.0%)	
TT	2 (1.0%)	1 (1.2%)	1 (2.2%)	1 (1.6%)	
Alleles					
C	354 (88.1%)	141 (84.9%)	77 (85.6%)	104 (83.9%)	
T	48 (11.9%)	25 (15.1%)	13 (14.4%)	20 (16.1%)	0.647
rs59693083					
Genotypes					
AA	139 (69.2%)	61 (73.5%)	28 (62.2%)	40 (64.5%)	
AG+GG	62 (30.8%)	22 (26.5%)	17 (37.8%)	22 (35.5%)	0.517
AG	56 (27.9%)	19 (22.9%)	16 (35.6%)	21 (33.9%)	
GG	6 (2.9%)	3 (3.6%)	1 (2.2%)	1 (1.6%)	
Alleles					
A	334 (83.1%)	141 (84.9%)	72 (80.0%)	101 (81.5%)	
G	68 (16.9%)	25 (15.1%)	18 (20.0%)	23 (18.5%)	0.750

The allele and genotype distributions of these SNPs in non-SIRS AP patients and SIRS patients are listed in Table 4. We found that, at rs5029924, SIRS patients more frequently harbored T alleles than did non-SIRS AP patients (22.3% vs. 12.9%; *p* = 0.028, OR: 1.936; 95% CI: 1.067–3.512), and minor allele T-carriers (CT+TT genotypes) were fewer among the non-SIRS AP patients than among the SIRS patients (24.5% vs. 42.6%; *p* = 0.018, OR: 2.286; 95% CI: 1.143–4.569). The allele and genotype frequencies of the rs59693083 polymorphism did not differ between SIRS and non-SIRS AP patients (all *p*>0.05). In addition, LD analysis demonstrated a lack of linkage disequilibrium between the two loci (D′ = 0.727 and r^2^ = 0.353).

**Table 4:** Genotype and allele frequencies of *TNFAIP3* promoter variants in AP patients with and without SIRS.

	Non-SIRS AP	SIRS	*p* value	OR	95% CI
rs5029924					
Genotypes					
CC	108(75.5%)	27(57.4%)			
CT+TT	35(24.5%)	20(42.6%)	0.018	2.286	1.143–4.569
CT	33(23.1%)	19(40.4%)			
TT	2(1.4%)	1(2.2%)			
Allele types					
C	249(87.1%)	73(77.7%)			
T	37(12.9%)	21(22.3%)	0.028	1.936	1.067–3.512
rs59693083					
Genotypes					
AA	98(68.5%)	31(66.0%)			
AG+GG	45(31.5%)	16(34.0%)	0.743	0.890	0.442–1.790
AG	44(30.8%)	12(25.5%)			
GG	1(0.7%)	4(8.5%)			
Allele types					
A	240(83.9%)	74(78.7%)			
G	46(16.1%)	20(21.3%)	0.249	0.709	0.395–1.274

AP indicates acute pancreatitis; CI indicates confidence interval; OR indicates odds ratio; SIRS indicates systemic inflammatory response syndrome.

### 3.4. Effect of the rs5029924 polymorphisms on promoter activity

**Figure 1:**
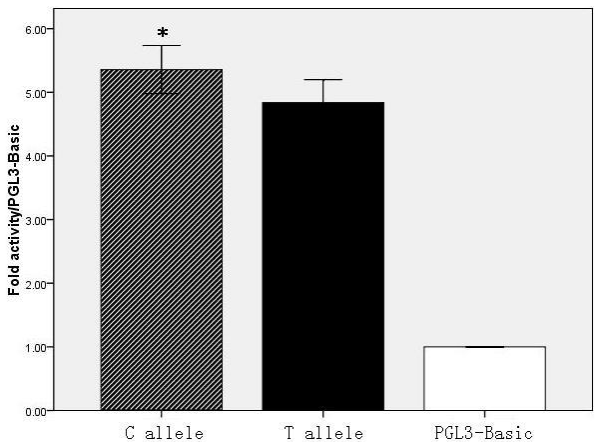
Effect of the *TNFAIP3* rs5029924 polymorphisms on transcription activity. Dual-luciferase activity was assayed in cells transfected with constructs bearing the C allele or the T allele of the rs5029924 *TNFAIP3* promoter variant. The firefly/*Renilla* luciferase activity ratio was normalized for transfection efficiency using a control plasmid, pRL-CMV. Results were expressed as fold-increase in firefly/*Renilla* luciferase activity of the *TNFAIP3* promoter construct vector as compared with pGL3-Basic. The firefly/*Renilla* luciferase activity of the C allele-construct was significantly higher than that bearing the T allele (*p* = 0.026). * *p*<0.05 compared with the T allele.

The possible functional significance of the rs5029924 polymorphisms was then examined by observing the effect of the alleles on the promoter activity of *TNFAIP3*. Figure 1 shows that the T variation at position −827 could significantly weaken the relative *Luciferase*/*Renilla* activities compared to the −827C allele (4.84±0.18 vs. 5.36±0.19, *p* = 0.026).

### 3.5. Analysis of association of *TNFAIP3* mRNA expression and protein levels with rs5029924

**Figure 2:**
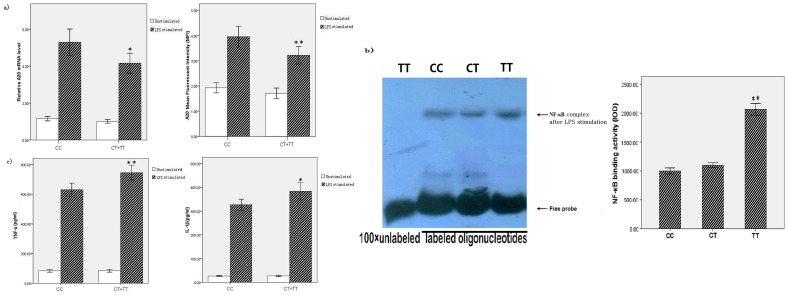
Effect of the rs5029924 polymorphism on in vitro LPS stimulation of whole blood. **a**) After LPS stimulation, mRNA and protein expression levels of TNFAIP3 were significantly different between individuals harboring the CC and individuals harboring the CT+TT genotypes (*p* = 0.012 and 0.007, respectively). **b**) NF-κB bound to specific DNA sequences were determined by electrophoretic mobility shift assays on polyacrylamide gels, after LPS stimulation. The IOD of NF-κB complex in individuals harboring the TT was significantly higher than the CC and TT genotype (*p*<0.001), and no significantly difference was recorded between the CC and TT genotype (*p* = 0.065). **c**) The TNF-α and IL-1β levels in supernatants were significantly different between individuals harboring the CC or the CT+TT genotypes after LPS stimulation (*p* = 0.001 and *p* = 0.011, respectively). * *p*<0.05, ** *p*<0.01 compared with the CC genotype.

Samples from 60 healthy people, including 30 with the CC genotype, 28 with the CT genotype, and 2 with TT genotype at rs5029924, were used in in vitro LPS stimulation experiments. Because the TT genotype was present at a low frequency, we combined the results of the minor allele T-carriers, i.e., individuals with the CT and TT genotypes, for analysis. Age and gender distribution were not significantly different between the carriers of the CC genotype and CT+TT genotypes. As shown in Figure 2a, data were calculated as relative levels, and bar graphs represented the averages of relative mRNA levels and A20 MFI for the 30 CC and 30 CT+TT subjects. No significant difference was observed between the *TNFAIP3* mRNA expression levels of the two groups in unstimulated peripheral blood (*p* = 0.056). After stimulating with LPS for 4 h, the *TNFAIP3* mRNA expression in peripheral blood was significantly higher in individuals with the CC genotype compared with those with the CT+TT genotypes (*p* = 0.012); more specifically, the *TNFAIP3* mRNA level in individuals with the CC genotype was 1.27-fold that of individuals with the CT+TT genotypes, by 2^−ΔΔCT^ analysis. A20 protein expression showed the same trend, in that no significant difference was seen between the genotype groups in the absence of stimulation (*p* = 0.135). Yet, with LPS stimulation, A20 protein expression was significantly higher in individuals with the CC genotype compared with those with the CT+TT genotypes (*p* = 0.007).

### 3.6. Analysis of association of NF-κB activity with rs5029924

PMBC nuclear extracts from individuals with different genotypes at rs5029924 were incubated with chemoluminescently labeled oligonucleotides corresponding to the consensus NF-κB-binding sequence, and the resulting complexes were resolved by PAGE. As shown in Figure 2b, all genotypes were capable of forming complexes, but the ability of NF-κB to bind to the DNA was strongest in individuals with the TT genotype, followed by those with the CT genotype, and weakest in those with the CC genotype, after LPS stimulation. The IOD of NF-κB complex in individuals harboring the TT was significantly higher than the CC and TT genotype (*p*<0.001), but no significant difference was recorded between the CC and TT genotype (*p* = 0.065). Competition experiments indicated that these complexes bound to the allelic variants in a sequence-specific manner.

### 3.7. Analysis of association of TNF-α and IL-1β levels with rs5029924

Figure 2c shows the relationship between the rs5029924 alleles and TNF-α and IL-1β production. After LPS stimulation, samples from subjects who were homozygous for the C allele of rs5029924 demonstrated lower levels of TNF-α and IL-1β compared with those carrying the CT+TT genotypes (*p* = 0.001 for TNF-α; *p* = 0.011 for IL-1β). However, no significant association was observed between TNF-α and IL-1β levels and genotypes at rs5029924 under the unstimulated conditions (*p* = 0.881 for TNF-α; *p* = 0.812 for IL-1β, respectively).

In addition, the expression of A20 and cytokines levels assays were also performed using SIRS samples, the results indicated CC genotypes had higher A20 expression and lower TNF-α, IL-1β levels compared with CT+TT genotypes (include this data as Data S1).

## Discussion

Factors underlying genetic predisposition for the development and progression of AP are largely unknown. Although several gene variants have been implicated in the susceptibility to and/or progress of AP [6]–[9], [26], [27], the effect of variants in genes involved in negative regulation of inflammation signaling pathways on AP has rarely been reported.

As a negative regulator of NF-κB pathways, the inhibitory action of A20 is an important mediator in the signal transduction involved in inflammation. Several studies have reported the relationship between A20 protein and pancreatic disease, such as pancreatic cancer, and the response to islet transplantation [28], [29]. NF-κB activation was also found to be enhanced in pancreatitis and in the systemic inflammatory response in both patients and animal models [30], [31]. Moreover, animal studies have shown that A20-deficient mice fail to regulate the TNF-α-induced NF-κB pathway, and that their inflammatory response spirals out of control, resulting in increased mortality [32]. Therefore, it is likely that AP is mediated, at least in part, by A20.

Although not previously tested in AP, variant alleles of *TNFAIP3* have been shown to affect the course of disease in other inflammatory conditions. Recent studies have revealed associations between *TNFAIP3* polymorphisms and psoriasis [33], rheumatoid arthritis [34], systemic lupus erythematosus [35], type 1 diabetes mellitus [36], type 2 diabetes mellitus [37], Behcet's disease [38], and celiac disease [39]. Moreover, Adrianto I et al. have reported the rs5029924 SNP has been shown to be associated with SLE in European and Korean populations [24].

This is the first report of the potential relationship between SNPs within the promoter of *TNFAIP3* and AP in Han Chinese patients. The two most informative SNPs studied here did not reveal any association with susceptibility to AP, but the rs5029924 polymorphism was shown to play a significant role in the progression of pancreatic inflammation to systemic inflammation in AP patients. There appeared to be no marked interaction between the two SNPs, because they were not in linkage disequilibrium. The MAF of the rs5029924 SNP in Utah residents with Northern and Western European ancestry from the CEPH collection (CEU) was 2.7%, while in Yoruba in Ibadan Nigeria (YRI) was 41.2%; this difference may play a significant role in susceptibility to acute pancreatitis in other populations.

Based on results of the above case–control study, the reason for the relationship of rs5029924 with SIRS in AP patients remains largely unknown. Chen et al. indicated that NF-κB-dependent excessive inflammation may contribute to the development and progression of AP [30]. Thus, the differences in the specific NF-κB binding to sequences containing the C or the T allele may be key to the spread of inflammation in AP patients. The relationship may also be caused by a functional non-synonymous SNP (F127C/rs2230926), which was in the same linkage block as rs5029924 [25]. We used LPS, an important inflammatory agent, to stimulate peripheral blood in vitro; this mimicked the process of an uncontrolled inflammatory reaction in vivo. The results supported the hypothesis that the rs5029924 polymorphism influences the expression of *TNFAIP3*, and leads to changes in the activity of NF-κB.

Taken together, the role of the rs5029924 polymorphism in progression from AP to SIRS may be as follows. As predicted by bioinformatics analysis, the T allele of rs5029924 affects the binding of GATA-1 and GATA-2, which could reduce *TNFAIP3* expression by changing promoter activity [40]. This then reduces the ubiquitination of signaling molecules in the NF-κB pathway, thereby modulating the pathway upstream of NF-κB activation and leading to increasing inflammatory cytokine levels that amplify the inflammatory cascade, finally contributing to the development of systemic inflammation and a severe clinical course of AP. However, several aspects of this proposed process remain to be confirmed; we plan to further investigate the effect of this variant on transcription factor binding and substrate ubiquitination.

The lack of influence of the rs59693083 polymorphism on AP may be explained by its location, which is remote from the transcription start site and the transcription factor-binding domain, so that it may only play a limited role, if any, on the promoter activity of *TNFAIP3*.

This study was limited by the relatively small sample size of AP patients, which may have exacerbated the limitations caused by the relatively low frequency of the minor allele. The association discovered in this study therefore also needs to be replicated in other institutions and in other populations.

In conclusion, our data indicated that the rs5029924 polymorphism in the *TNFAIP3* promoter site may be considered as an important genetic risk factor for susceptibility to post-AP SIRS in the Han Chinese population. This effect may be mediated by changes in NF-κB activity caused by variable expression of *TNFAIP3*. Our finding could be of value in evaluation of the risk of developing SIRS in AP patients, and could eventually lead to individualized targeted treatment in AP patients, based on genetic background.

## Supporting Information

Data S1 **The expression of A20 and cytokines levels in SIRS samples with different genotypes.**The PBMC and plasma samples from SIRS patients were detected the expression of A20 and cytokines levels, and CC genotypes had higher A20 expression and lower TNF-α, IL-1β levels compared with CT+TT genotypes.
(XLS)
